# Identifying key players in dark web marketplaces through Bitcoin transaction networks

**DOI:** 10.1038/s41598-023-50409-5

**Published:** 2024-01-29

**Authors:** Elohim Fonseca dos Reis, Alexander Teytelboym, Abeer ElBahrawy, Ignacio De Loizaga, Andrea Baronchelli

**Affiliations:** 1 The Alan Turing Institute, London, NW1 2DB UK; 2 Department of Mathematics, City, University of London, London, EC1V 0HB UK; 3 Department of Economics, University of Oxford, Oxford, OX1 3UQ UK; 4 Chainalysis Inc, New York, NY USA; 5 PayPal Inc, San Jose, CA USA; 6 UCL Centre for Blockchain Technologies, University College London, London, WC1E 6BT UK

**Keywords:** Complex networks, Applied physics, Statistical physics, thermodynamics and nonlinear dynamics

## Abstract

Dark web marketplaces have been a significant outlet for illicit trade, serving millions of users worldwide for over a decade. However, not all users are the same. This paper aims to identify the key players in Bitcoin transaction networks linked to dark markets and assess their role by analysing a dataset of 40 million Bitcoin transactions involving the 31 major markets in the period 2011–2021. First, we propose an algorithm that categorizes users either as buyers or sellers, and show that a large fraction of the trading volume is concentrated in a small group of elite market participants. We find that the dominance of markets is reflected in trading properties of buyers and sellers. Then, we investigate both market star-graphs and user-to-user networks, and highlight the importance of a new class of users, namely ‘multihomers’, who operate on multiple marketplaces concurrently. Specifically, we show how the networks of multihomers and seller-to-seller interactions can shed light on the resilience of the dark market ecosystem against external shocks. Our findings suggest that understanding the behavior of key players in dark web marketplaces is critical to effectively disrupting illegal activities.

## Introduction

The dark web has been home to many unregulated online commercial platforms facilitating the trade of illicit goods^1–10^. This ecosystem, composed of the dark web marketplaces (DWMs) and the network of user-to-user (U2U) transactions^11–13^, has proven to be sensitive to changes in demand for goods and services and resilient against external shocks^5,7,14,15^. Despite the risks associated with their illegal nature, millions of users have traded on these platforms since the launch of Silk Road, the first modern DWM^16^. The popularity of DWMs stems from users being able to access them easily and anonymously, and trade items that are not available in regulated markets. Owing to their unregulated character, DWMs offer no formal protection to buyers and sellers. Many DWMs were closed, either by law enforcement operations or by exit scams, leaving their users with significant losses^1^. This uncertainty has not prevented this ecosystem from growing due to its ‘Whack-a-Mole’ dynamics: when a market is closed, users migrate to an alternative platform in a swift and coordinated fashion^7,15^.

Surprisingly, although DWMs have gained significant attention from the scientific community and law enforcement agencies, little is known about the key players sustaining their unusual adaptability and responsive dynamics. Several papers have uncovered essential phenomena related to DWMs. However, owing to the difficulty of identifying relevant transactions, most studies rely on user surveys^17,18^ and data scraped from DWM websites^19–24^. In particular, these studies are based on user reviews which carry many inaccuracies, for instance, with respect to the time and value of the transaction^19^, that further compound error in other measures. Moreover, data scraped from the DWMs cannot assess the U2U transactions which account for the largest fraction of the total trading volume of the ecosystem^13^.

Conversely, transaction networks obtained from the blockchain contain the entire transaction data of the DWMs and U2U transactions, allowing a thorough investigation of the ecosystem as a whole. In fact, previous studies on DWM transaction networks have revealed crucial aspects of the ecosystem^13–15^. However, they have so far mainly focused on DWM *users*, without distinguishing between buyers and sellers, and neglecting the different weight that more active users may have in the system. The reason is that the operational structure of DWMs inherently hides the seller–buyer link, as all transactions are made through the marketplace. Buyers send money to the marketplace, which in turn sends the money to the seller. Thus, further analyses in this direction have been hindered by the lack of heuristics able to identify these two key classes of actors in transaction networks and their roles in the structure and dynamics of the ecosystem.

Here, we set out to find the main actors in the DWM ecosystem and assess their systemic impact on a dataset of 40 million Bitcoin transactions involving the 31 major markets in the period 2011–2021. First, we propose a simple algorithm to identify buyers and sellers. Importantly, the algorithm returns reasonable estimates for the number of sellers when compared against a benchmark of nine DWMs where estimates exist. Then, we reveal a concentration of activity around an elite group of participants, where a large fraction of the trading volume is driven by a small number of players. Specifically, we uncover distinct types of buyers and sellers based on their activity between markets and the U2U network, and detect a shift in the ecosystem’s activity towards the U2U network after a major external shock in the markets. We find that trading properties of buyers and sellers reflect the dominance of DWMs in the ecosystem. By formally describing the ecosystem of DWMs as a temporal network where nodes are the entities and directed edges are transactions pointing from source to destination, we consider different networks of buyers and sellers, promoting different functions in the ecosystem. In particular, we analyse networks of ‘multihomers’, defined as users that are simultaneously trading in multiple markets. We show that these users play a crucial role in the connectivity of the ecosystem because they act as connectors between markets. Analogously, we identify and characterise ‘multisellers’ (i.e., multihomers that are sellers) and ‘multibuyers’ (i.e., multihomers that are buyers). Furthermore, we analyse the seller-to-seller (S2S) network, i.e., the network composed only of transactions among sellers, which can be regarded as a supply chain network of illicit goods and services. We highlight that these networks exhibit different resilience regimes in the presence of external shocks, the ecosystem’s resilience being mostly guaranteed by the network of buyers rather than sellers.

## Results

### Buyers and sellers

**Figure 1:**
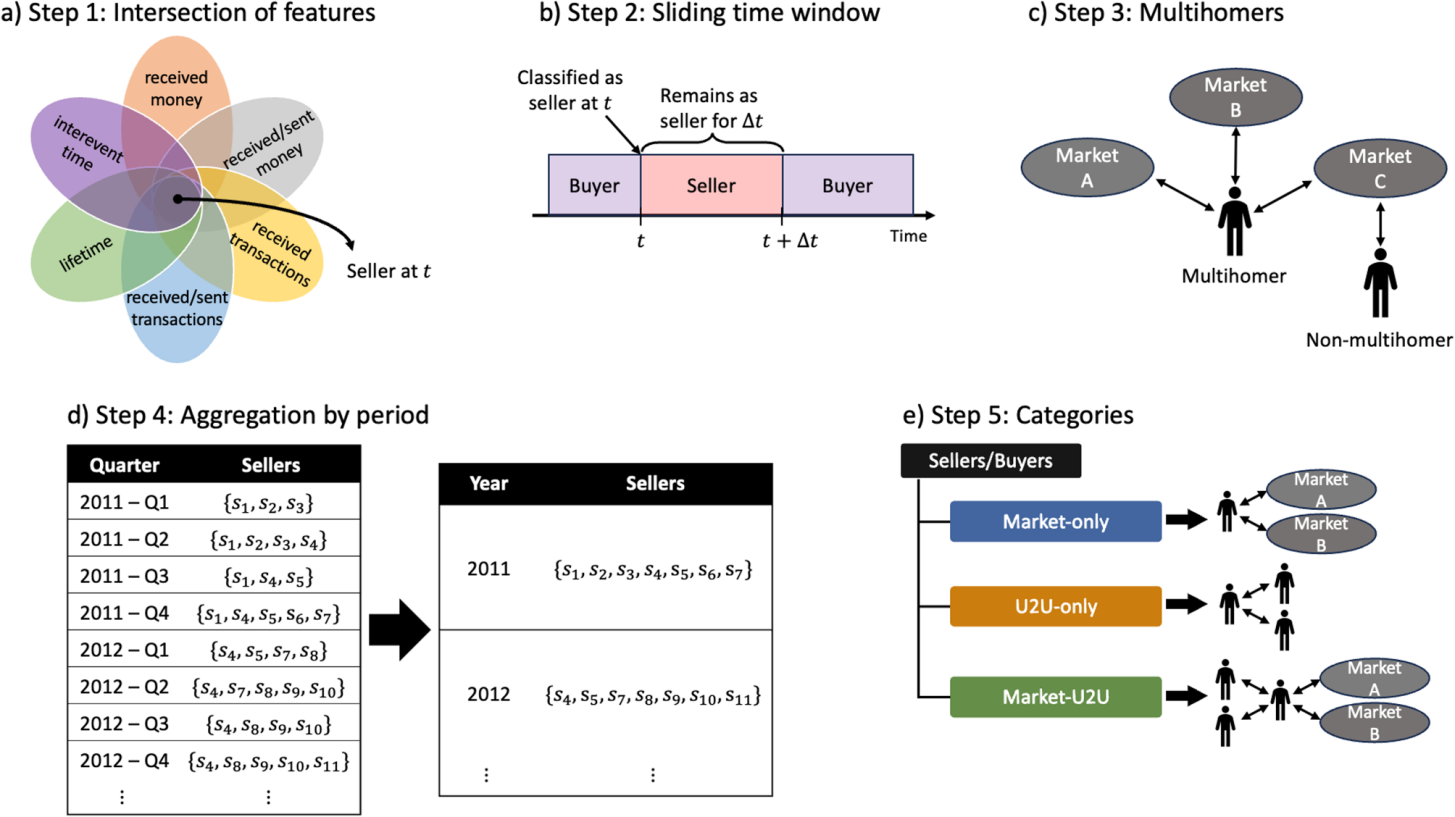
The five steps of the classification of entities as buyer and sellers. (**a**) Step 1: Intersection of features—an entity is classified as a seller at time *t* when they satisfy the six features simultaneously. (**b**) Step 2: Sliding time window—when an entity is classified as a seller, they remain as a seller during $\Delta t$ days. (**c**) Step 3: Multihomers—we identify sellers and buyers that are simultaneously active in more than one market. (**d)** Step 4: Aggregation by period—we aggregate the lists of obtained sellers and buyers according to a given calendar period. (e) Step 5: Categories—buyers and sellers are divided into the three categories.

To characterize the structure and dynamics of the ecosystem of DWMs, we start by classifying all traders either as buyers or sellers. We implement a method of classification based on exchanged money, number of transactions, and time activity for each entity, as illustrated in Fig. 1 (see “Methods” for details). Our method identifies sellers, whereas buyers are entities which are not classified as sellers. We consider each market separately, i.e., we obtain a time series of buyers and sellers for each market, and we use the same method and classification parameters in the U2U network. Therefore, an entity can be classified as a seller in one or more markets and/or the U2U network simultaneously. We feed the classification process with seven parameters: minimum money received (denoted by *M*), minimum ratio between received and sent money (denoted by $\alpha$), minimum number of transactions (denoted by *T*), minimum ratio between number of received and sent transactions (denoted by $\beta$), minimum lifetime (denoted by *L*), maximum mean interevent time of transactions (denoted by $\tau$), and a sliding time window (denoted by $\Delta t$).

We set $M = 100$ USD, $\alpha = 3$, $T = 10$, $\beta = 3$, $L = 10$ days, $\tau = 10$ days, and $\Delta t = 30$ days in the rest of the paper. The classification method is robust with respect to the values assigned to the classification parameters, and returns reasonable estimates compared against a benchmark of nine markets (see Supplementary Information Section S1). It is worth noting that parameters were set conservatively in order to avoid false positives in the classification of sellers. As a consequence, our method returns generally fewer sellers than other estimates^25–33^.

**Figure 2:**
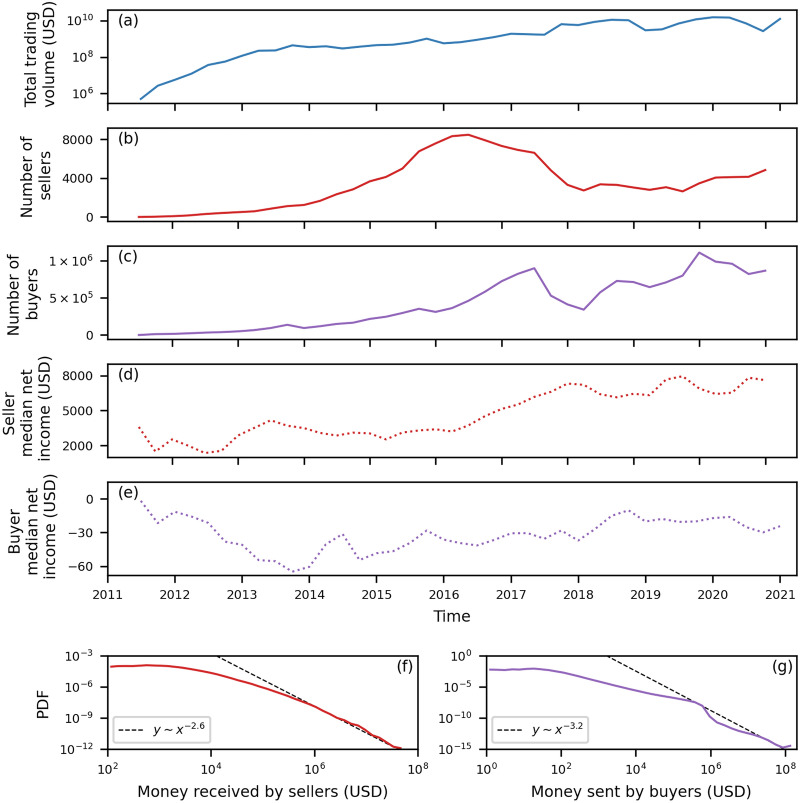
Evolution of buyers and sellers in the ecosystem of DWMs. We consider the whole ecosystem, i.e., all markets and the U2U network. In panel (**a**), we show the total quarterly trading volume in USD. In panels (**b, c**), we show the number of all sellers and buyers per quarter, respectively. In panels (**d, e**), we show the median net income in USD of all sellers and buyers per quarter, respectively. In panels (**f, g**), we show the PDF of the total money received by each seller and the total money sent by each buyer, respectively.

The evolution of the ecosystem of all buyers and sellers obtained from the considered markets and the U2U network is shown in Fig. 2. The total quarterly trading volume is shown in Fig. 2a. Although it shows fluctuations, including those caused by external shocks, the ecosystem exhibits a positive growth trend in terms of trading volume.

Our classification shows that the number of sellers is significantly smaller than the number of buyers, as shown in Figs. 2b,c, respectively. The number of actors in the ecosystem is affected by several factors, especially market closures. Notably, the number of buyers and sellers significantly drops after the operation Bayonet in the last quarter of 2017, which shut down AlphaBay and Hansa markets, causing a major shock in the ecosystem^34^. However, the number of buyers rapidly recovers, which does not happen to sellers.

To compare the financial behaviour between sellers and buyers, we compute the median net income, defined as the median of the difference between the cumulative money received and sent from all transactions (in all markets and U2U network) made by each entity as a function of time, as shown in Figs. 2d,e, respectively. The median net income is positive for sellers while negative for buyers throughout the whole period of observation. This result is not trivial because, although the classification induces a positive net income for sellers, it is performed on each market and the U2U network separately, while the median net income is computed based on all transactions made by an entity. In fact, when we compute the total net income for each seller, a considerable fraction (16%) has a negative net income because they spend in markets where they are not classified as sellers, or in the U2U network. Moreover, we find a change of trend between the seller and the buyer median net income time series which reflects the dominance of markets, as detailed in the next section.

To study the distribution of the trading volume between users, we analyse the total money received and sent by each user. We find that 5% of sellers—those who have received more than $2.3 \times 10^5$ USD—account for 81% of the total amount of money received by all sellers. Conversely, we find that 5% of buyers—those who have sent more than $2.3 \times 10^4$ USD—account for 92% of the total amount of money sent by all buyers. Therefore, there is small fraction of actors responsible for moving most of the trading volume in both directions, i.e., buying and selling. We observe this concentration of trading volume in the probability density functions (PDFs) of the total money received by each seller and the total money sent by each buyer, as shown in Figs. 2f,g, respectively. In both cases, we see a significant heterogeneous distribution, with estimated tail exponents equal to $-2.6$ ($R^2 = 0.999$) for sellers and $-3.2$ ($R^2 = 0.996$) for buyers, hence showing similar behaviours quantitatively (see Supplementary Information Section S2 for details of the fitting procedure).

### Dominant markets

**Figure 3:**
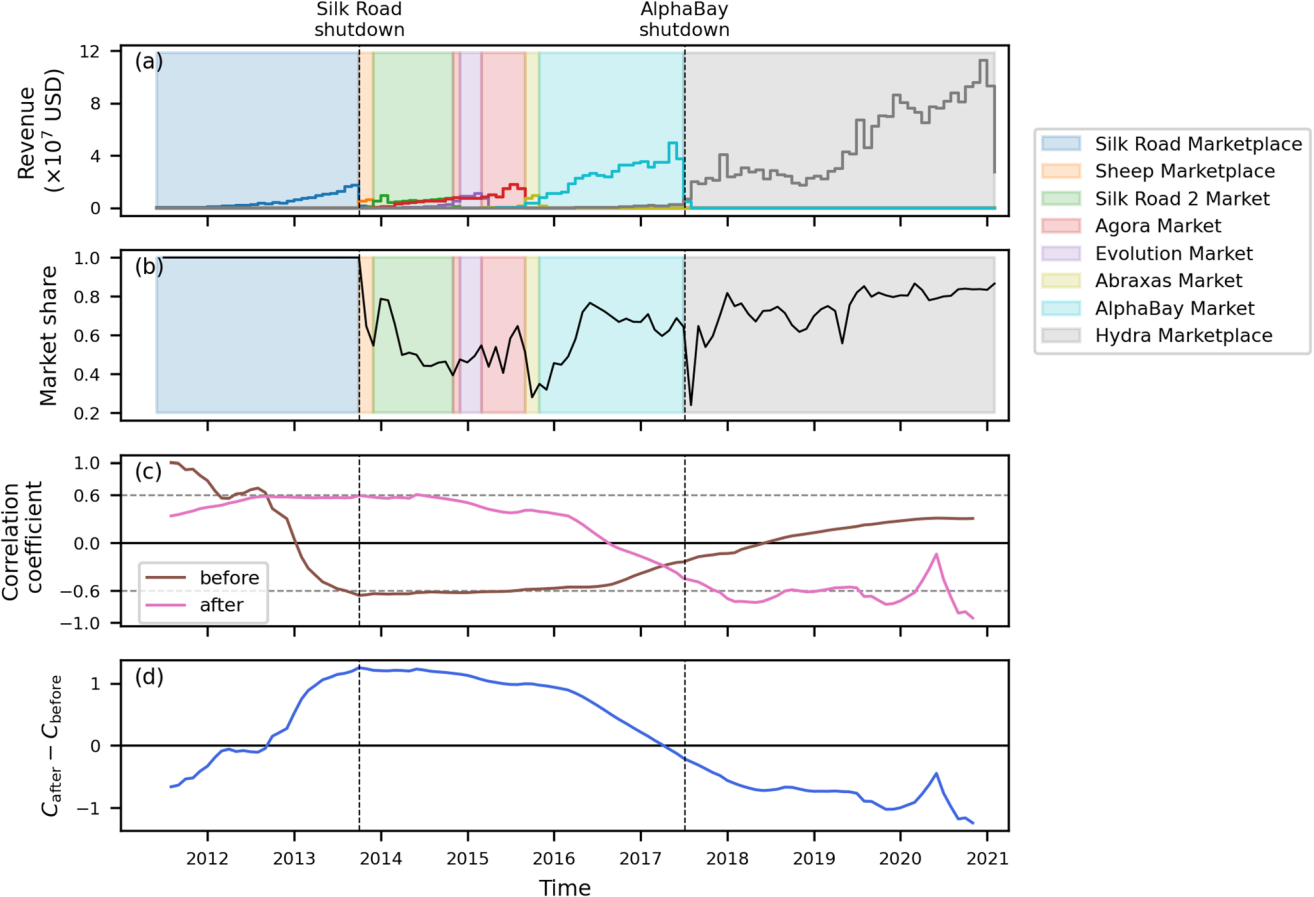
Structural change in the ecosystem by dominant markets. (**a**) The monthly dominant market by revenue in USD. (**b**) The monthly market share of the corresponding dominant market. (**c**) The two time series of the correlation coefficient between the time series of seller monthly median net income and buyer monthly median net income before and after each month. In other words, for each month, we calculate two values of correlation coefficient, one for the seller and buyer median net income time series before that month ($C_\text{before}$), and another for the time series after that month ($C_\text{after}$). (**d**) The time series of the difference $C_\text{after} - C_\text{before}$. In all panels, the first and the second vertical dashed lines represents the time of Silk Road’s and AlphaBay’s shutdown, respectively.

Throughout the period of observation, there were eight dominant markets, as shown in Fig. 3. In accordance with sector reports^1,4,8–10^, we measure the dominance in terms of revenue in USD, i.e., the dominant market is the market with the largest revenue, as shown in Fig. 3a. We assess the strength of a market’s dominance by the market share (i.e., the market revenue divided by the sum of the revenues of all markets) and by the length of the time interval the market remains dominant, as shown in Fig. 3b. In other words, a strong dominance is characterized by a large market share ($>0.5$) kept for a long period. The ecosystem was initially strongly dominated by Silk Road. After Silk Road shutdown, there was a transition period when no market was dominant. Then, by the end of 2015, AlphaBay became the dominant market until its takedown by operation Bayonet^34^. Following AlphaBay shutdown, Hydra emerged as the dominant market exhibiting the strongest observed dominance to date^8–10^. Three markets consistently sustain over 60 percent market share, namely Silk Road, AlphaBay, and Hydra.

The period of dominance by Silk Road is unique, because the ecosystem structure is effectively composed of and dominated by a single market, expressed by a market share equal to one, as shown in Fig. 3b. After the shutdown of Silk Road, in the last quarter of 2013, the ecosystem evolves to a structure where several markets coexist. This structural change is reflected in the median net income of sellers and buyers, as shown in Fig. 2d,e. While the curves for the seller and buyer median net income were negatively correlated before Silk Road’s shutdown, after that moment they became positively correlated. Specifically, sellers show a trend of increase and buyers a trend of decrease in their median net income before the shutdown.

To corroborate this switch of trend, first, we compute the Pearson correlation coefficient between the seller and buyer time series of the monthly median net income before and after each month. In other words, for each month, we calculate two correlation coefficients, one between the two time series before that month ($C_\text{before}$), and another between the time series after that month ($C_\text{after}$), as shown in Fig. 3c. We observe that, around the time of Silk Road’s shutdown, the two time series are negatively correlated before (i.e., $C_\text{before}<0$), and positively correlated after (i.e., $C_\text{after}>0$). Then, we further calculate the difference between the two correlation coefficient time series, i.e., $C_\text{after} - C_\text{before}$, as shown in Fig. 3d. The maximum value of $C_\text{after} - C_\text{before}$ occurs in the month prior to Silk Road’s shutdown, indicating the change in the ecosystem’s structure. Interestingly, we find the situation is reverted in the last four years, when the ecosystem is strongly dominated by Hydra. Although Hydra is not a single dominant market as Silk Road was, its dominance is marked by a high market share, consistently staying above 80% in the last two years. During this period, we observe $C_\text{after} < 0$ and $C_\text{after} - C_\text{before}$ reaches its minimum.

These results suggests that a strong dominance in the ecosystem induces anticorrelation between the median net income of sellers and buyers. In fact, if we split the whole period of observation into three epochs—before the shutdown of Silk Road ($t < t_{SK}$), after the shutdown of AlphaBay ($t > t_{AB}$), and the period in between ($t_{SK}< t < t_{AB}$)—and calculate the coefficient of correlation between the seller and buyer median net income time series for each epoch, we find that $C_{t < t_{SK}} = -0.66$ (p value $= 1.4 \times 10^{-4}$), $C_{t > t_{AB}} = -0.47$ (p value $= 1.7 \times 10^{-3}$), and $C_{t_{SK}< t < t_{AB}} = 0.31$ (p value $= 4 \times 10^{-2}$).

### Multihomers and categories

To analyse the connectivity of the whole ecosystem, i.e., how markets are connected with each other, we consider sellers and buyers that are simultaneously active on multiple platforms. We refer to these users as “multihomers”. In particular, multihomers that are sellers in multiple markets are *multisellers*, and similarly for buyers we have the *multibuyers*. Specifically, to be classified as a multiseller, a user must be classified as a seller in at least two markets simultaneously. Likewise for multibuyers. The multihomers play a crucial role in the ecosystem because they act as edges between markets. While there may occasionally be multihomers who are active in several markets simultaneously (see Supplementary Information Section S3), multihomers predominantly operate in at most two markets throughout the period of observation. Hence we do not distinguish multisellers by the number of markets in which they operate.

Additionally, three mutually exclusive categories of sellers (buyers) naturally emerge:

1. Market-only sellers (buyers), who are active in one or more markets and not in the U2U network;
2. U2U-only sellers (buyers), who are active only in the U2U network, and
3. Market-U2U sellers (buyers), who are simultaneously active in both markets and the U2U network.

**Figure 4:**
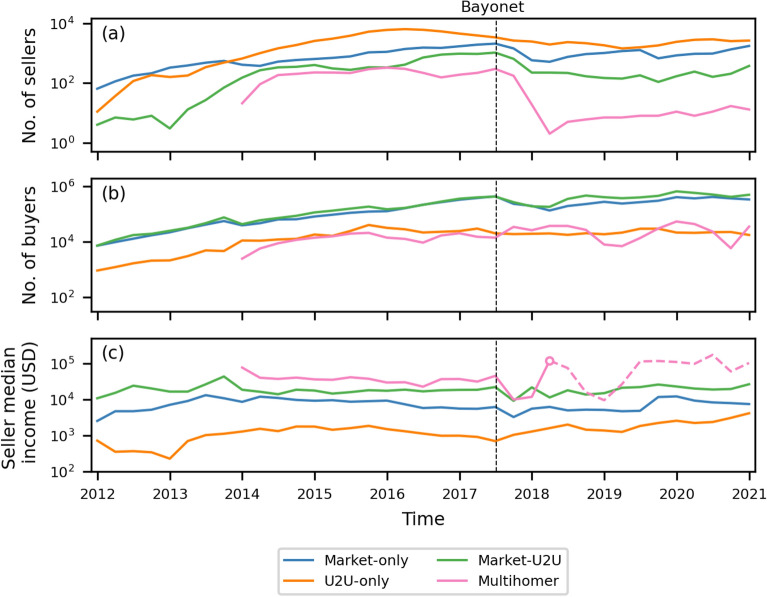
The evolution of the different types of sellers and buyers. (**a**) The number of sellers for each category and multisellers per quarter. (**b**) The number of buyers for each category and multibuyers per quarter. (**c**) The quarterly median net income of sellers. The empty point on the multiseller median income line demarcates the quarter with only two multisellers. After that quarter, their number remains small, which is represented by the dashed line. In all panels, the dashed vertical line marks the time of operation Bayonet.

The number of sellers in each category and multisellers as a function of time is shown in Fig. 4a. Until the end of 2013, when Silk Road is the dominant market (see Fig. 3), market-only sellers is the dominant category, and there are no multisellers. From the last quarter of 2013, U2U-only sellers become the largest category of sellers and remains as the largest throughout the rest of the observation period. The large number of U2U-only sellers is in accordance with previous results that showed that the trading volume in the U2U network is significantly larger than that of DWMs^13^ (also see Supplementary Information Figure S8).

With the advent of several markets at the beginning of 2014, the number of multisellers rapidly grows, representing more than 20% of all sellers until the beginning of 2016 (see Supplementary Information Section S3). During 2016 and 2017, AphaBay becomes the dominant market (see Fig. 3), polarizing sellers around its own ecosystem, such that the fraction of multisellers decreases to 10% of all sellers until its closure. Then, after operation Bayonet, the number of sellers in all categories and multisellers significantly drops, as shown in Fig. 4a. Notably, the number of multisellers suffers the largest drop of $-99\%$ by the end of the first quarter of 2018. Interestingly, while the other categories of sellers show signs of recovery relative to their previous levels, the number of multisellers remains low after that shock (see Supplementary Information Section S4).

The results for buyers are different, as shown in Fig. 4b. Throughout the whole period of observation, the dominant category of buyers is market-U2U buyers followed by market-only buyers, representing on average 52% and 42% of all buyers, respectively. The U2U-only category is comparatively small, representing only 6% of all buyers on average. The number of market-U2U and market-only buyers also drops as a consequence of operation Bayonet. However, compared to sellers, the drop is notably smaller, and the number of buyers rapidly recovers to previous values. On the other hand, the number of U2U-only buyers is less affected. Moreover, the number of multibuyers increases, which suggests a fast response from buyers to external shocks by trying to diversify their sources.

To study the performance of sellers, we analyse the quarterly median income, i.e., the quarterly median of the money received by each seller, for each category and multisellers, as shown in Fig. 4c. We find that multisellers have the largest median income throughout the period of observation—except in the last quarter of 2017 and 2018, when they have the second largest median income. They are followed by market-U2U sellers, then market-only sellers, and lastly U2U-only sellers. Therefore, although larger in number, U2U-only sellers typically make the smallest income. This suggests that sellers with more diverse sources of income, such as multisellers and market-U2U sellers, are able to produce a higher income. Additionally, we observe that, except for U2U-only sellers, the median income of the other types of sellers drops after the major shock caused by operation Bayonet (see Supplementary Information Section S4). The most affected are multisellers, with a drop of 78% in the median income, followed by market-U2U and market-only sellers, with a drop of 59% and 47%, respectively. Although these three types of sellers are significantly affected, they recover and surpass the median income value they had before Bayonet (see Fig. 4c; Supplementary Information Section S4).

**Figure 5:**
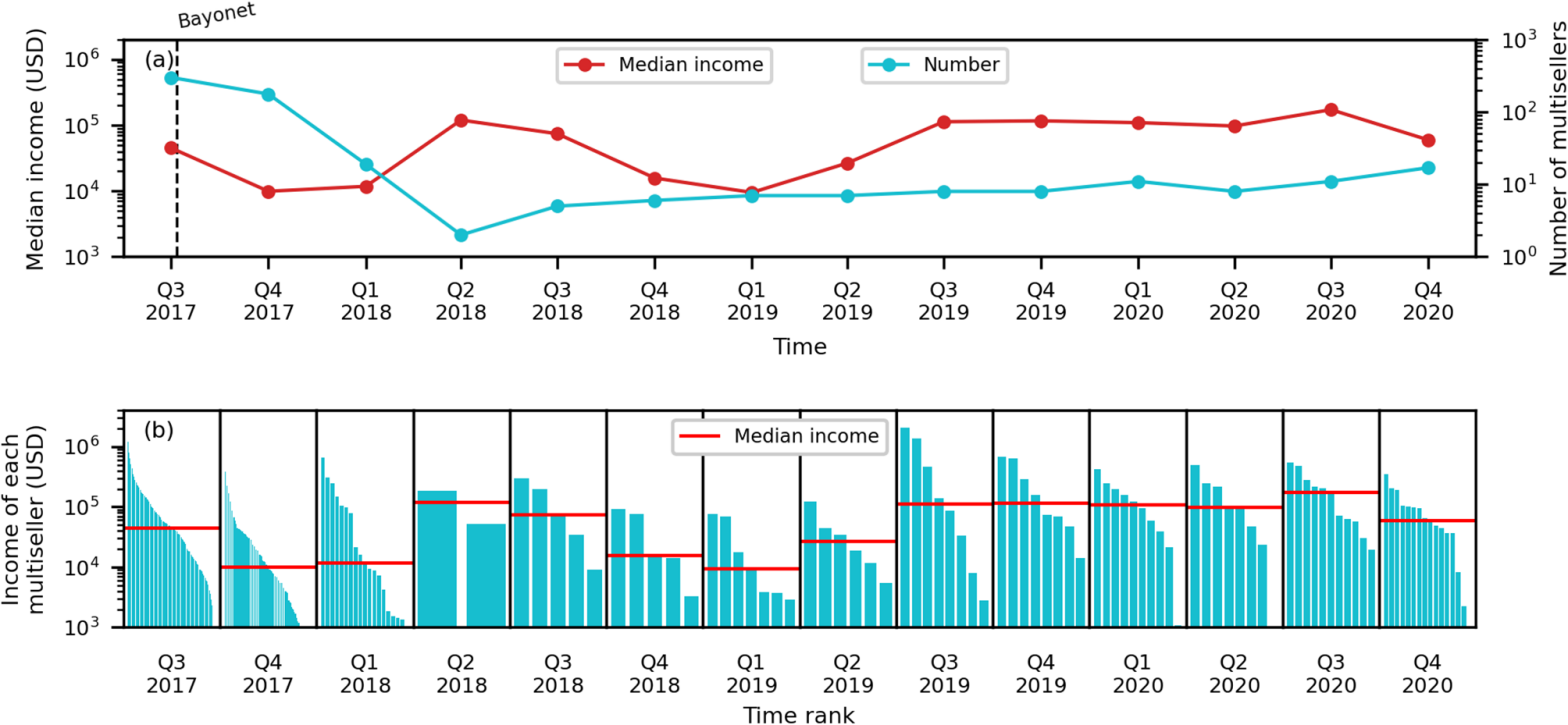
The number of multisellers steeply decreases after operation Bayonet but they still sustain high incomes. (**a**) The quarterly median income in USD and number of multisellers. The dashed vertical line marks the time of operation Bayonet. (**b**) The ranked quarterly income in USD of each multiseller. The horizontal red line is the median income for each quarter.

Notably, the statistical significance of the high median income of multisellers after operation Bayonet is low, because their number plummets—only two survive by the end of the second quarter of 2018—and, although with a trend of increase, it remains considerably small, as shown in Figs. 4c and 5a. Nevertheless, by analysing the income of each multiseller, we observe that the median income still reflects their high income, as shown in Fig. 5b. Individually, they are able to yield significant high incomes compared to other types of sellers.

In contrast to the other types of sellers, the median income of U2U-only sellers increases after operation Bayonet. Moreover, we observe a trend of increase in their median income relative to the value before operation Bayonet—an increase of almost six times by the end of the period of observation (see Supplementary Information S4). This indicates a shift in the ecosystem towards the U2U network. Further supporting this interpretation, we observe that the trading volume of the U2U network increases after Bayonet, while the trading volume of markets decreases (see Supplementary Information Section S4).

These critical changes may not be perceived from macroscopic measures of the ecosystem as a whole. For instance, the typical net income of sellers is seemingly unaffected, as shown in Fig. 2d, akin to what is observed in Fig. 2a, where the overall ecosystem volume quickly recovers after market closures. This is an outcome of the ecosystem’s resilience, largely supported by the migration of users^15^. Correspondingly, the multihoming activity is a mechanism that contributes to the ecosystem’s resilience. Because they are already active in more than one market, the migration cost for the multihomers is usually smaller compared to that for non-multihomer users, especially for sellers, that need to rebuilt their reputation^23^.

### Multiseller network

**Figure 6:**
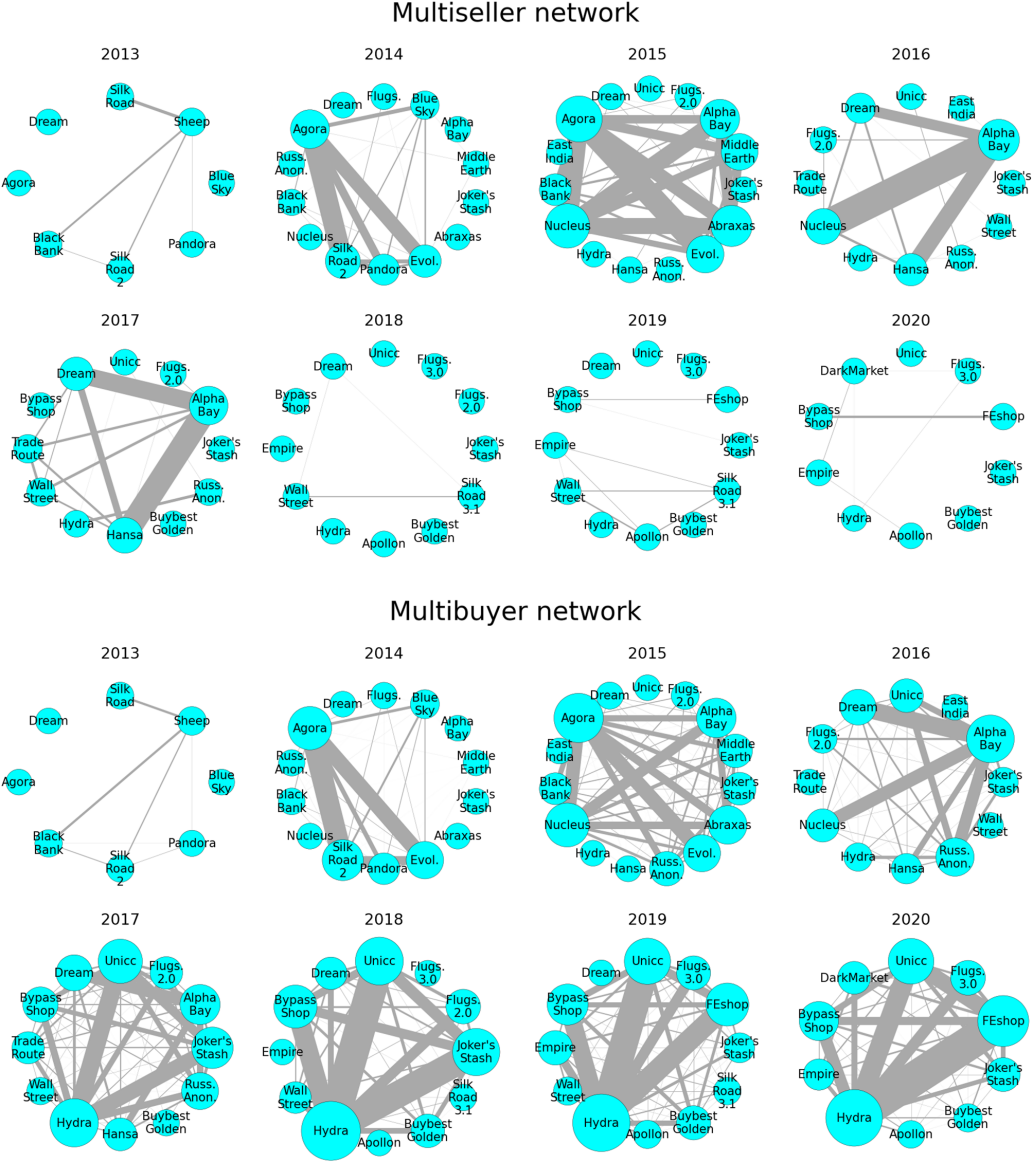
The structural change in the multiseller network and the resilience of the multibuyer network. Temporal network of multisellers (top) and multibuyers (bottom) between markets for each year. Nodes are markets that are active during the year. Edges are multihomers, i.e., traders that are simultaneously active in both markets (sellers in the multiseller network, and buyers in the multibuyer network). The width of the edges is proportional to the number of multihomers acting between the markets.

To investigate the role of sellers on the broader ecosystem of DWMs, we consider a temporal network where nodes are the active markets (i.e., markets in operation at the time), and an edge between the nodes represents the number of multisellers between them, what we henceforth call the *multiseller network*. The evolution of the multiseller network is shown in Fig. 6. Until 2012, there is only one active market, namely Silk Road market, and hence no multihomer activity. From 2013 until 2015, the multiseller network grows in terms of connectivity, showing an increasing number of edges spread across different markets. During 2016 and 2017, the edges are polarized by AlphaBay, the dominant market (see Fig. 3). Then, between 2017 and 2018, there is a drastic structural change in the multiseller network structure due to operation Bayonet, after which the connections almost vanished. This change persists until the end of the observed period of the data set (also see Supplementary Information S4).

Although the number of multisellers suffers a severe drop (Fig. 4a) and the multiseller network drastically shrinks (Fig. 6), the net income of multisellers remains persistently the largest among sellers throughout the whole period of observation, as shown in Fig. 4. This suggests that the multiseller activity is sensitive to external shocks but also that it yields higher profits.

### Multibuyer network

Buyers simultaneously active on multiple markets also play the role of connectors in the ecosystem. Therefore, we analyse the temporal network where nodes are the active markets and an edge between the nodes represents the number of multibuyers between them, what we henceforth call the *multibuyer network*. The structural change seen in the multiseller network is not observed in the multibuyer network, as show in Fig. 6. The evolution of the multibuyer network follows a similar pattern to the multiseller network until 2015, despite a stronger polarization around Hydra instead of AlphaBay during 2017. However, after the operation Bayonet, although the network shows a decrease in connectivity, it still remains highly connected and with a large number of active multibuyers. Moreover, the network had already fully recovered by 2019 showing a strong resilience against external shocks.

### Seller-to-seller network

In order to investigate the role of direct transactions between market participants, we now analyse the evolution of the S2S network, i.e., the network of the U2U transactions involving only sellers. The nodes of the S2S network are active sellers (i.e., sellers that are trading at the time) and two sellers are connected by an edge if at least one transaction was made between them during the considered snapshot period. Although the S2S network is composed only of U2U transactions, all categories of sellers (i.e, market-only, U2U-only, and market-U2U) are present in the S2S network. For instance, market-only sellers are entities classified as sellers only in markets, but that may promote U2U transactions with other sellers, hence being part of the S2S network. Therefore, the S2S network can be seen as a proxy for a distribution network of illegal products.

To reduce the presence of noise in the S2S network, we consider only stable U2U pairs, i.e., pairs that have at least three transactions throughout the whole period of observation^13^. The trading volume generated by stable pairs is more than five times larger than that of non-stable pairs^13^. The S2S network is mostly populated by U2U-only sellers, followed by market-only, and market-U2U (see Supplementary Information Section S5).

**Figure 7:**
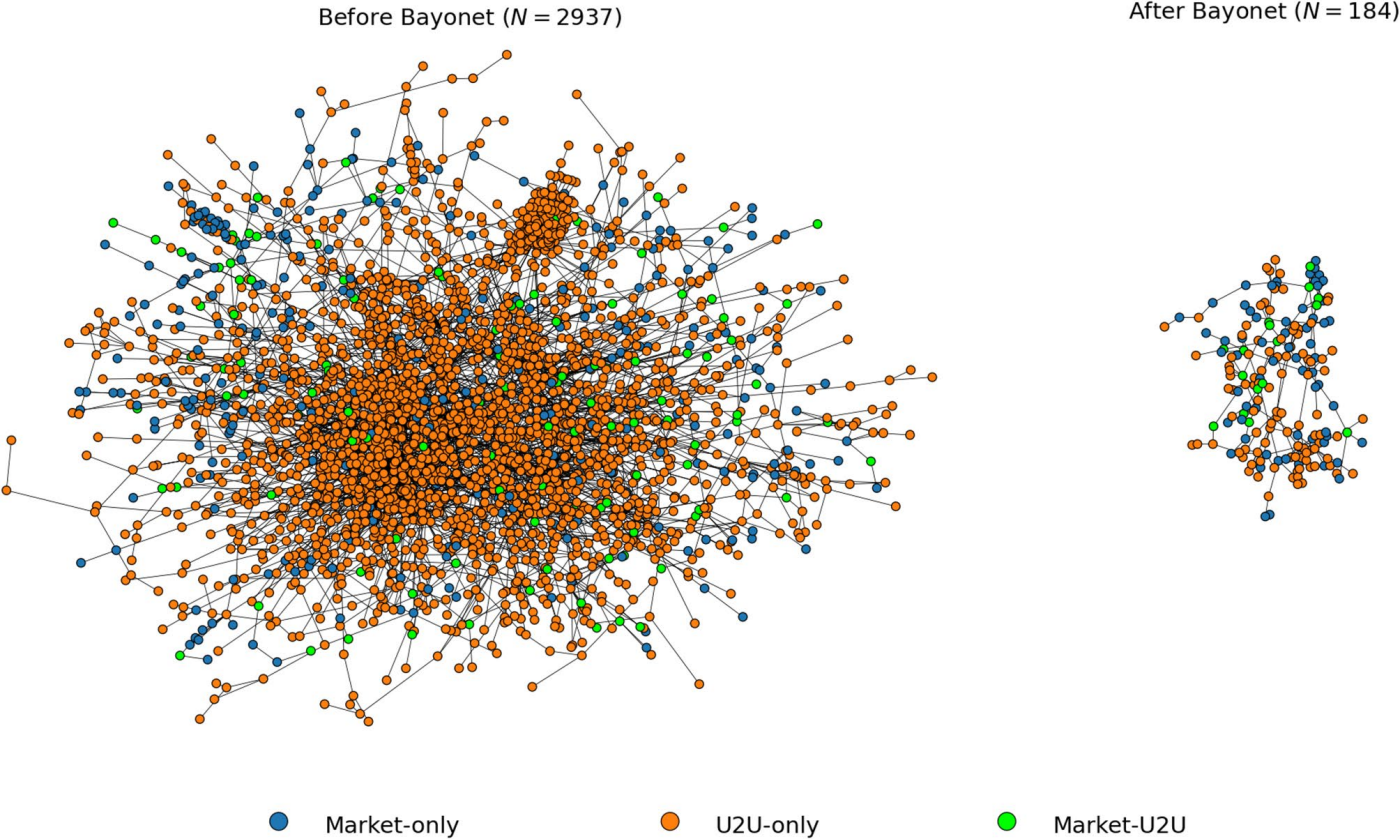
The impact of the operation Bayonet on the S2S network. The largest component of the S2S network one year before and one year after the operation Bayonet. Nodes are sellers that are active within the time period, and an edge is placed between two sellers if at least one transaction occurs between them during the period. The S2S network is mostly populated by U2U-only sellers.

In Fig. 7, we show the largest component of the S2S network one year before the operation Bayonet and one year after. The network shows a notable structural change, significantly shrinking. However, the evolution of the S2S network shows a different pattern than that observed in both the multiseller and the multibuyer networks.

**Figure 8:**
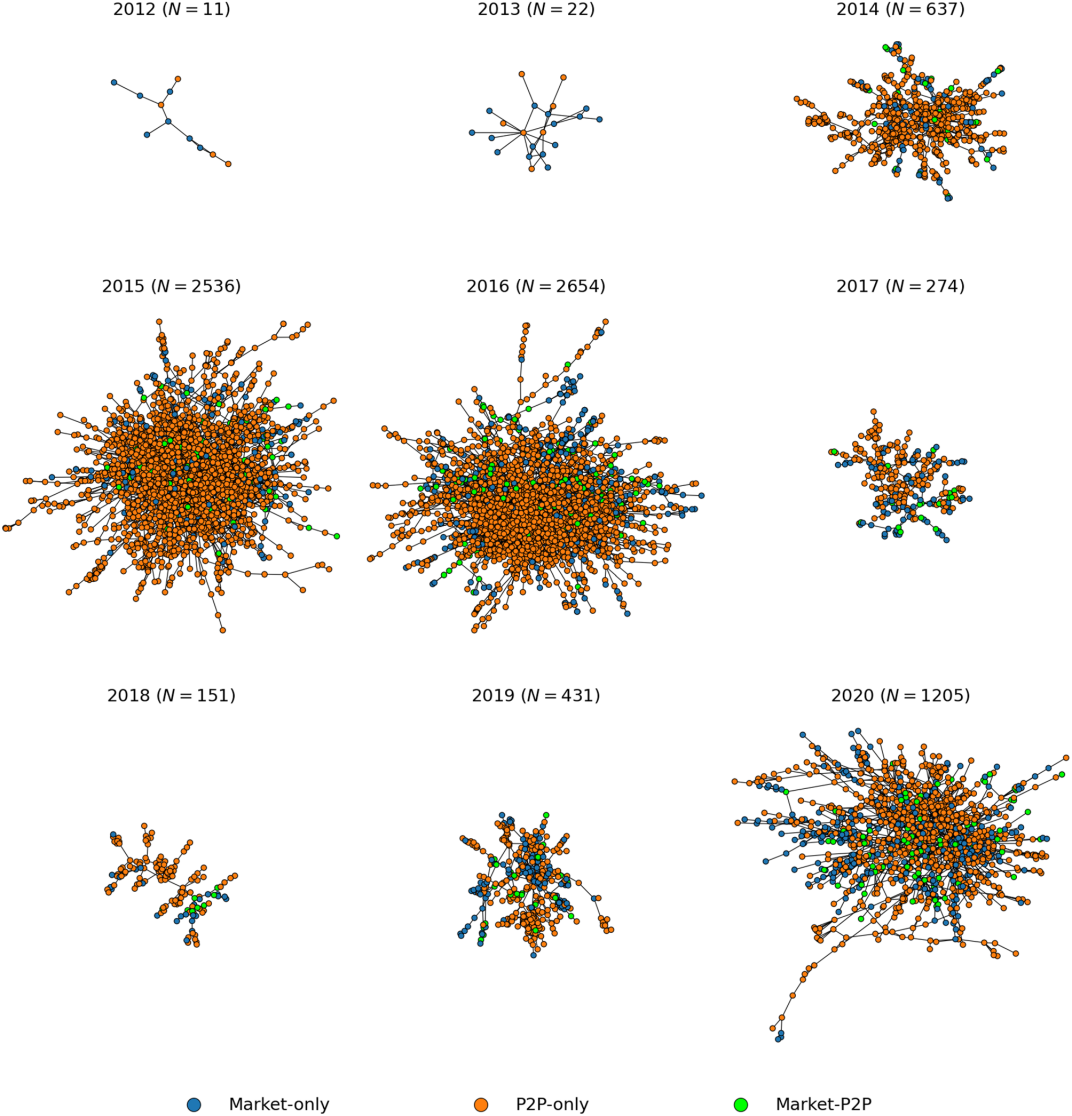
The intermediate resilience regime of the S2S network. The largest component of the S2S network of U2U transactions between sellers for each year with the respective number of nodes (*N*). The nodes are sellers that are active in that year, and an edge is placed between two sellers if at least one transaction occurs between them during that year. The network is mostly populated by U2U-only sellers, followed by market-only sellers. After a major external shock in 2017, the S2S network shrinks but, unlike the multiseller network, recovers, and grows again (though slower than the multibuyer network).

From 2012 to 2016, the largest component of S2S network continuously grows in number of nodes and connections, as shown in Fig. 8. Then, during 2017 and 2018, it shows the structural change due to operation Bayonet, when it shrinks. However, unlike the multiseller network, the S2S network recovers during 2019 and 2020, but slower than the multibuyer network recovery. Therefore, the S2S network appears to be more resilient than the multiseller network but less than the multibuyer network. The same pattern is observed in the whole S2S network (see Supplementary Information Section S5).

## Methods

### Data preprocessing

DWMs are illegal unregulated commercial websites. However, trading behaviour in DWM closely resembles what is observed on regulated online platforms despite their significant differences in operational and legal nature^14^. Nevertheless, due to their unregulated nature, DWMs exhibit behaviours not observed in regulated marketplaces. They offer anonymity to their users by using and developing specialized tools. DWMs are accessed through darknet browsers supporting the onion routing protocol (e.g., Tor), which provides anonymous communication connections^35^. Additionally, transactions are made with cryptocurrencies, mostly Bitcoin, which also provide anonymity to the transaction parties^6,36^. While the Bitcoin blockchain is publicly available on Bitcoin core^37^ or other third-party APIs such as *Blockchain.com*^38^, a market or a user can generate a new address for each transaction. To track the transactions of markets and users as entities, the data need to be pre-processed in order to map groups of addresses into entities.

We use data of DWM transactions on the Bitcoin blockchain pre-processed by Chainalysis Inc. Although other coins are used, such as Monero recently, Bitcoin is still the mostly used in the ecosystem, being supported by more than 93% of markets^7,9^. The pre-processing relies on established state-of-the-art heuristics to cluster addresses into entities, such as cospending, intelligence-base, and behavioral clustering^39–42^. The resulting data set includes for each transaction the source and destination entities, the time, and the value of the transaction.

**Figure 9:**
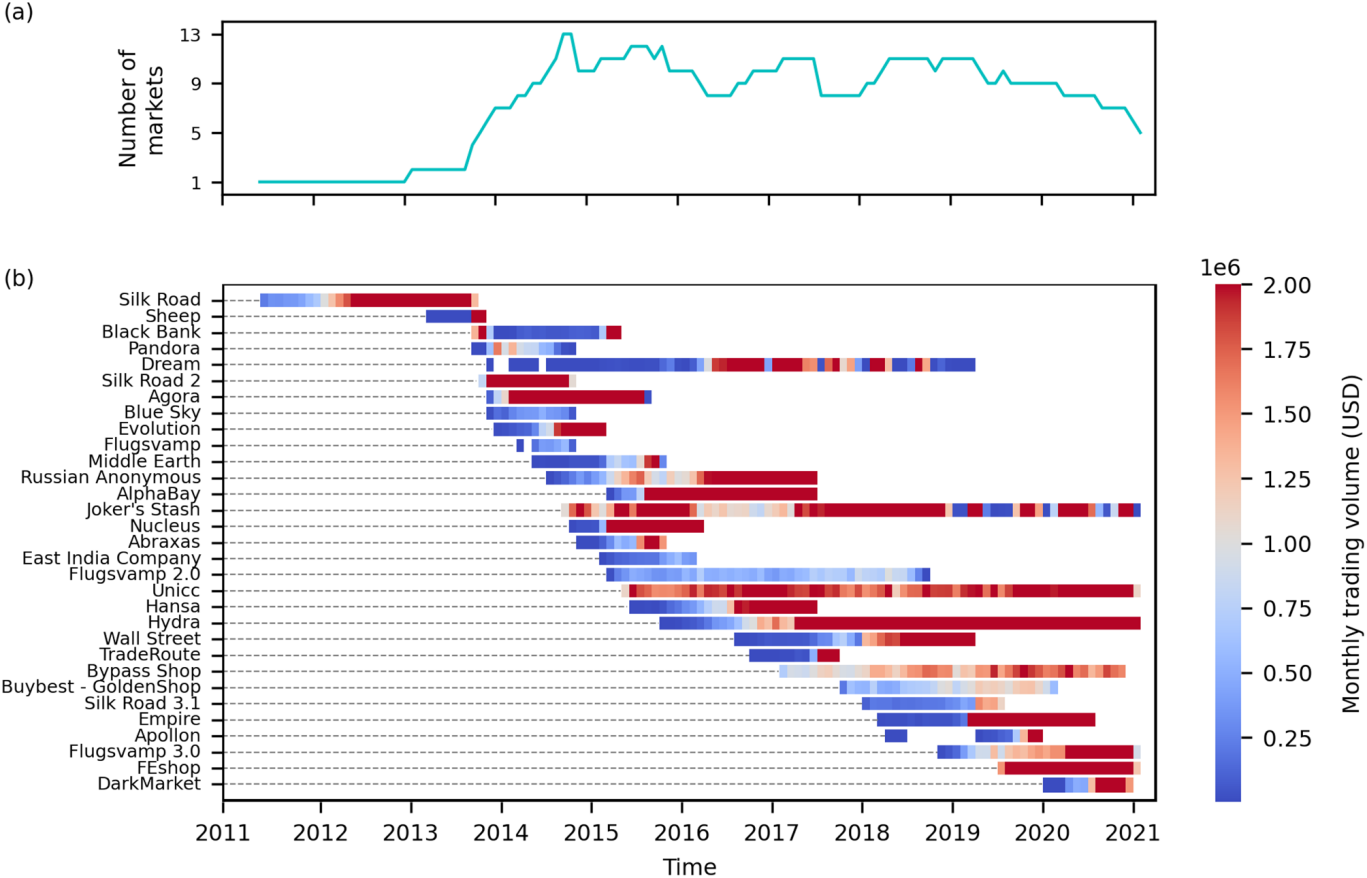
The evolution of the ecosystem of DWMs. (**a**) Total monthly number of active markets. (**b**) Lifetime of markets. The horizontal bars represent each market lifetime, i.e., the time when the market becomes active until its closure, and is colored according to the market’s monthly trading volume in USD. In the vertical axis, markets are in the chronological order of their launch date, although for some markets the activity effectively starts after the launch date (e.g., AlphaBay).

We exclude transactions with a value larger than $5 \times 10^5$ USD and smaller than 0.01 USD. To include the major marketplaces and obtain statistically relevant measures, we selected markets with an average daily trading volume larger than 15,000 USD and a lifetime larger than six months. As a result, our data set consists of more than a decade of the entire transaction history of 31 DWMs between June 2011 and February 2021, as shown in Fig. 9.

### Network structure of transactions

We represent the network of transactions by temporal networks where nodes are entities (markets and users), and directed edges represent a transaction pointing from the source to the destination entity and endowed with the time and value of the transaction. Each marketplace is a star-graph where the central node is the marketplace, and the leaf nodes, i.e., the first-neighbors, are the marketplace users. Therefore, all transactions involving the market have the market either as a source or as a destination node.

Additionally, we analyse the U2U network of transactions, i.e., the transactions between pairs of market first-neighbors where the source and destination nodes are market users without the market as an intermediate. In the U2U network, an edge connects nodes that are not necessarily users of the same market. Therefore, the U2U network connects different market star-graphs. Previous studies have shown that, although the number of users and transactions is larger in markets, the trading volume in the U2U network is larger than that of markets^13^.

### Classification of sellers and buyers

We classify all entities either as sellers or buyers as a function of time. The result is a time series of lists of sellers and buyers for each period and for each market and the U2U network. The classification is performed in five steps (see Fig. 1), as detailed next.

Step 1: Intersection of features. We use six features to classify sellers: (1) received money, (2) ratio between received and sent money, (3) number of received transactions, (4) ratio between number of received and sent transactions, (5) lifetime, and (6) mean interevent time of transactions, as defined next.

The classification is a function of time. For each entity, we keep track of the cumulative values of the six features for each transaction performed by the entity over time. Then, we assign a threshold value for each feature. To be classified as a seller, an entity must simultaneously satisfy the six feature threshold criteria, as follows.

First, we consider the total cumulative received money by each entity *i* at time *t*, denoted by $m^\text{rec}_i(t)$. This feature is satisfied when $m^\text{rec}_i(t) \ge M$, where *M* is the fixed value of minimum total cumulative received money.

Second, we consider the ratio between the total cumulative received and sent money by each entity *i* at time *t*, denoted by $R_i(t)$, i.e., $R_i(t) \equiv m^\text{rec}_i(t) / m^\text{sent}_i(t)$, where $m^\text{sent}_i(t)$ is the total cumulative sent money by entity *i* at time *t*. This feature is satisfied when $R_i(t) \ge \alpha$, where $\alpha$ is a constant value.

Third, we consider the total cumulative number of received transactions by each entity *i* at time *t*, denoted by $n_i^\text{rec}(t)$. This feature is satisfied when $n^\text{rec}_i(t) \ge T$, where *T* is the fixed value of minimum total cumulative number of received transactions.

Fourth, we consider the ratio between the total cumulative number of received and sent transactions by each entity *i* at time *t*, denoted by $Q_i(t)$, i.e., $Q_i(t) \equiv n^\text{rec}_i(t) / n^\text{sent}_i(t)$, where $n^\text{sent}_i(t)$ is the total cumulative number of transactions sent by entity *i* at time *t*. This feature is satisfied when $Q_i(t) \ge \beta$, where $\beta$ is a constant value.

Fifth, we consider the lifetime of each entity *i* at time *t*, defined as the time interval between the first and the last transaction performed by the entity until time *t*, denoted by $\ell _i(t)$. This feature is satisfied when $\ell _i(t) \ge L$, where *L* is the fixed value of minimum lifetime.

Sixth, we consider the cumulative mean interevent time for each entity *i* at time *t*, defined as the mean of the sequence of time interval between consecutive transactions of an entity until time *t*, which we denote by $\phi _i(t)$. This feature is satisfied when $\phi _i(t) \le \tau$, where $\tau$ is the maximum value of mean interevent time.

Finally, given a set of parameters $\{M, \alpha , T, \beta , L, \tau \}$, we classify an entity *i* as a seller if $m^\text{rec}_i(t) \ge M$, $R_i(t) \ge \alpha$, $n^\text{rec}_i(t) \ge T$, $Q_i(t) \ge \beta$, $\ell _i(t) \ge L$, and $\phi _i(t) \le \tau$ are simultaneously satisfied at time *t*. Conversely, because the classification is a function of time, an entity that has been classified as a seller at time *t*, might lose the seller status if, at a future time, the features are not satisfied. This step is performed separately for each market and the U2U network. After classifying each entity according to its time series of transactions, we aggregate sellers daily, i.e., we obtain a daily time series of lists of sellers for each market and the U2U network.

Step 2: Sliding time window. The method used in step 1 captures the activity of entities in a continuous-time framework, i.e., the features are computed for each transaction taken by each entity. This results in a time series of sellers where sellers are irregularly classified because of oscillations on each entity specific activity, such as having a less frequent number of transactions during a period. Therefore, we use a sliding window of $\Delta t$ days to classify sellers, i.e., every day that an entity is classified as a seller, it remains as a seller for $\Delta t$ days, including the first day. After using the sliding time window for sellers, all entities that are not classified as sellers are classified as buyers for each day. At the end of step 2, we generate a daily time series of sellers and buyers for each market and the U2U network.

Step 3: Multihomers. The daily time series of sellers obtained in step 2 is a list of sellers for each day for each market. To identify multisellers, we first compute, for each pair of simultaneously active markets, the intersections of the daily lists of sellers obtained from step 2. Then, to obtain the daily time series of multisellers, we compute the union of the daily intersections of sellers between pairs of markets. We perform the same procedure to compute the daily time series of multibuyers but using the daily time series of buyers obtained from step 2.

Step 4: Aggregation by period. To observe the behavior of the ecosystem on specific calendar periods, such as weekly or quarterly, we select a time period and aggregate the daily time series through step 3 accordingly. For example, to obtain a monthly time series of sellers, we compute the union of the lists of sellers for each month. This step is independent of the sliding time window in step 2. For instance, if an entity is classified as a seller for 20 consecutive days in a month in Step 1 and $\Delta t = 30$ days, that entity will remain as a seller for 30 days from the last day of the 20 days, hence still being a seller in the next month. Therefore, at the end of step 4, we obtain a time series of buyers and sellers for each market and the U2U network according to the selected time period.

Step 5: Categories. For each period of time obtained in step 4, some sellers are active only in markets, others in the U2U network, or in both. Therefore, for each time period, we divide the sellers into three mutually exclusive categories: (1) market-only sellers, which are the union of sellers that are active in one or more markets but only markets and not in the U2U network; (2) U2U-only sellers, which are the union of sellers that are active only in the U2U network; and (3) market-U2U sellers, which are the union of sellers who are active in one or more markets and also active in the U2U network. For instance, multisellers belong to set of market-only or market-U2U sellers, but not to the set of U2U-only sellers by definition. Analogously, we divide buyers for each time period into three mutually exclusive categories: market-only buyers, U2U-only buyers, and market-U2U buyers. Specifically for buyers, when we compute the union or intersection of sellers across markets and the U2U network, we remove entities that are sellers in any market or the U2U network in that time period.

## Discussion

In this paper, we proposed a method for classifying users as sellers or buyers in the ecosystem of DWMs. We then identified three key categories of buyers and sellers that play different roles in the ecosystem: market-only traders, that are active only in markets but not in the U2U network; U2U-only traders, that are active only in the U2U network; and market-U2U traders that are active both in the markets and the U2U network simultaneously. Additionally, we singled out the multihomers, i.e., users that are simultaneously active in multiple markets, acting either as sellers (the multisellers), or as buyers (the multibuyers).

We showed that a small fraction of traders is responsible for a large fraction of the trading volume, and by analysing the networks of buyers and sellers, we found different resilience regimes. Shocks tend to induce serious structural changes in the multiseller network, but impact the multibuyer network much less severely. Interestingly, the S2S network shows an intermediate level of resilience, which suggests that the S2S network might play the role of a supply chain network on the dark web. Furthermore, after a shock, the activity of buyers is resumed almost immediately, while the activity of sellers recovers more slowly. These different regimes suggest that the ecosystem’s resilience is mainly supported by the high demand of buyers rather than the response of the sellers.

Despite consistent results, this study has limitations that may be addressed in future work. First, while the dataset is preprocessed with state-of-the-art methods, there is no ground truth for validation, and this uncertainty propagates to our findings. For instance, we cannot verify if an entity classified as seller is in fact a seller. Similarly, there is no unique choice for the classification parameters or ground truth for fitting them. In light of this, we have chosen the parameters conservatively, obtaining estimates for the number of sellers that are in general smaller than the ones produced by other methods. Second, our approach does not explicitly classify buyers, which are entities that were not classified as sellers. There is a gray zone in which some sellers and buyers may not be easily distinguishable in transaction networks. For instance, there may be sellers that make a small amount of transactions, or spend more than receive, which we would classify as buyers. Despite consistent results, this clearly leaves space for refinements. Nevertheless, it is important to stress that the results are robust under considerable variation of the parameters, indicating that the coherent picture emerging from our analysis does not depend on the details of the method. Future work may further extend the approach presented here, for example using machine learning methods to capture further behavioral regularities. Third, at any given moment we classify entities as either buyers or sellers. Yet it is possible that multiple roles are played at once. For example, in some cases, a seller in a given market may behave as a buyer in a second market or in the U2U network. This multi-role classification, to be implemented in future work, can help gain a more nuanced understanding of the ecosystem and the structure of the dark web supply chains.

Overall, our study provides a first step towards a better microscopic characterisation of the DWM ecosystem, indicating a direction of investigation that may be of interest to both researchers and law enforcement agencies. The results further support the recent efforts of law enforcement agencies to focus on individual sellers^43–45^, as well as, more recently, also buyers^46,47^. Since the beginning of DWMs’ activity, there has been a shift in the law enforcement approach from focusing on market admins towards sellers and buyers^9,13^. For instance, a recent London Metropolitan Police (MET) investigation examined the transactions of a seller profile on a DWM^10^. The investigation uncovered a local criminal organization linked to a large international drug supply operation. Therefore, key actors in the ecosystem of DWMs may play important roles in broader criminal networks. The finding that multisellers and, in specific cases, multibuyers play a central role in connecting the ecosystem, thus contributing to its resilience, may illuminate how to better target future law enforcement operations. In general, by understanding the operation of key players within the DWM ecosystem, our work highlights how appropriate strategies can be designed to counteract the online trade of illicit goods more effectively.

### Supplementary Information

Supplementary Information.

## Data Availability

All data needed to evaluate the conclusions in the paper are present in the paper. Additional data related to this paper may be requested from the corresponding author.
