# Supplementary material for: Identifying key players in dark web marketplaces through Bitcoin transaction networks
Source: Sci Rep. 2024 Jan 29;14:2385. doi: 10.1038/s41598-023-50409-5 (PMC10824757; doi:10.1038/s41598-023-50409-5)
Supplement: Supplementary file 1 — Supplementary Information. [file 41598_2023_50409_MOESM1_ESM.pdf]

Supplementary Information for: Identifying key players in dark web marketplaces through Bitcoin transaction networks

Elohim Fonseca dos Reis[1,2], Alexander Teytelboym[3], Abeer ElBahrawy[4], Ignacio De Loizaga[5], and Andrea Baronchelli[1,2,6,*]

[1] *The Alan Turing Institute, London NW1 2DB, UK*
[2] *Department of Mathematics, City, University of London, London EC1V 0HB, UK*
[3] *Department of Economics, University of Oxford, Oxford OX1 3UQ, UK*
[4] *Chainalysis Inc, New York, NY, USA*
[5] *PayPal Inc, San Jose, CA, USA*
[6] *UCL Centre for Blockchain Technologies, University College London, London WC1E 6BT, UK*
[*] *Corresponding author: andrea.baronchelli.1@city.ac.uk*

S1 Robustness of classification

Our classification method has seven parameters, namely the set of six features for classifying sellers, $\{M, \alpha, T, \beta, L, \tau\}$, and the size of the sliding time window Δt. To test the robustness of the classification with respect to the parameters, we run stress-tests where we vary one of the parameters independently and keep the other six fixed. In the following, when varying one of the parameters, we set the other parameters with the same values used in the main text, i.e., $M = 100$ USD, $\alpha = 3$, $T = 10$, $\beta = 3$, $L = 10$ days, $\tau = 10$ days, and $\Delta t = 30$. The classification under these values generates basal sets of buyers and sellers that are already conservative. Therefore, by varying the parameter values under these strict conditions, we induce extreme cases for the robustness stress-tests.

The set of buyers is effectively unaffected by the change of parameters, unless extreme values are used. For instance, if we use negative values in the first step of the classification. Therefore, we focused on variations caused in the set of sellers.

We performed robustness tests on markets and the U2U network separately because they have different aspects. For instance, a seller in the U2U network is a single vendor shop, while a seller in a DWM is part of a marketplace with many sellers. Most importantly, the time frame of the U2U network is the whole observation period, while each market has its own time frame which is the market lifetime hence much shorter than the U2U network time frame.

First, we analyse each of the six features used for classifying sellers in step 1 of the classification (see Methods in the main text). In Figure S1, we show the obtained time series of sellers for all markets when varying each of the six features. Overall, the results show that the classification is robust with respect to the features for markets, yielding the same qualitative results with a relative small variation. Specifically for the minimum number of transactions [Fig. S1(c)], the classification shows a larger variation compared to the other features because it selects more sellers for a small number of T. We also observe a larger variation during 2016 and 2017 when varying the minimum lifetime [Fig. S1(e)]. During this period, AlphaBay is the largest market which may attract several market sellers but that do not stay active for a long period.

The classification is also qualitatively robust in the U2U network, as shown in Fig. S2. However, the variation is larger in the U2U than markets for β and τ. Because the time frame of the U2U is the whole period of observation, it is more unlikely that users keep a small mean interevent time in the long term. The large variation on β may be associated to the fact that, compared to markets, there are less transactions in the U2U network, and U2U users may have a larger number of outgoing number transactions. Therefore,

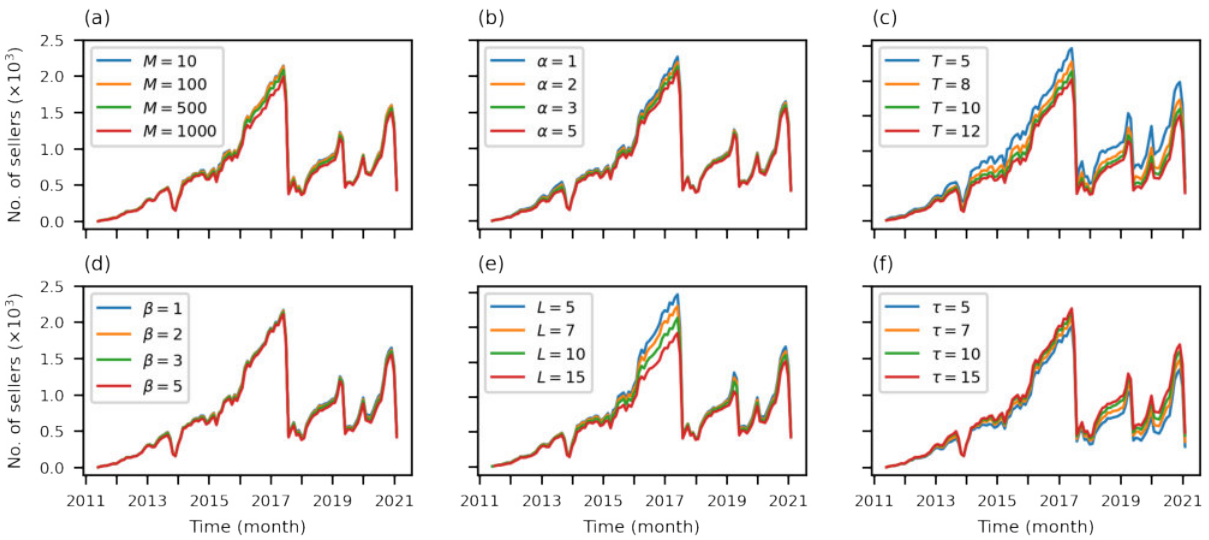

Figure S1: Robustness of the classification with respect to the six features for markets. We vary each feature independently (i.e., we vary one feature and keep the others fixed) and run the whole classification for all markets. (a) Obtained number of sellers when $M = 10$, 100, 500, and 1000 USD. (b) Obtained number of sellers when $\alpha = 1$, 2, 3, 4, and 5. (c) Obtained number of sellers when $T = 5$, 8, 10, and 12 transactions. (d) Obtained number of sellers when $\beta = 1$, 2, 3, and 5. (e) Obtained number of sellers when $L = 5$, 7, 10, and 15 days. (f) Obtained number of sellers when $\tau = 5$, 7, 10, and 15 days.

one may find reasonable to set different values to the six features used for the U2U network than those used for markets. However, we kept the same values across markets and the U2U network to consistently classify sellers, grasping the same behaviour in both situations.

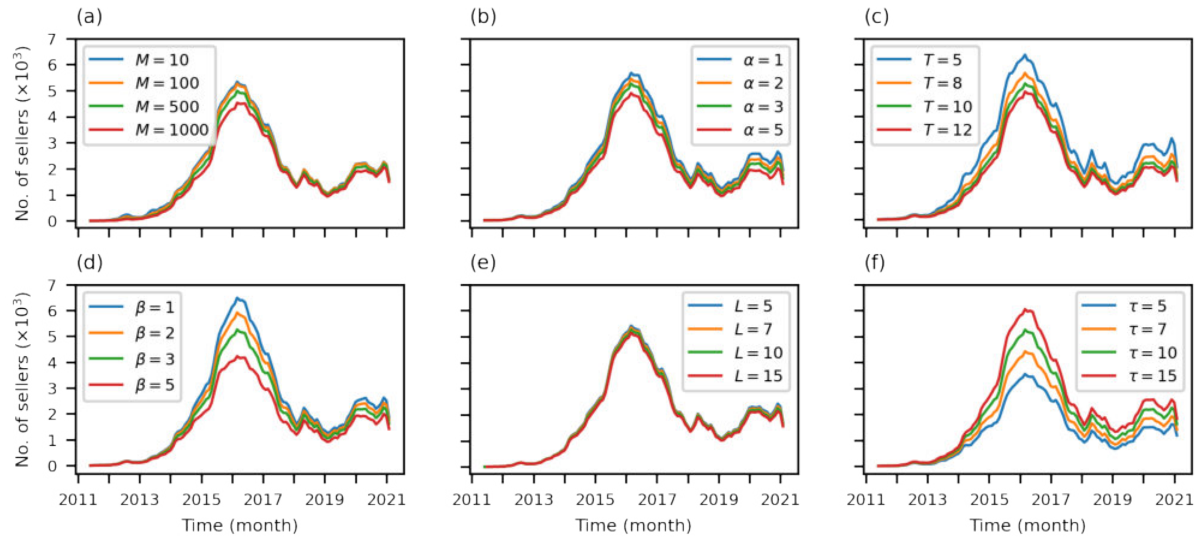

Figure S2: Robustness of the classification with respect to the six features for the U2U network. We vary each feature independently (i.e., we vary one feature and keep the others fixed) and run the whole classification for the U2U network. (a) Obtained number of sellers when $M = 10$, 100, 500, and 1000 USD. (b) Obtained number of sellers when $\alpha = 1$, 2, 3, 4, and 5. (c) Obtained number of sellers when $T = 5$, 8, 10, and 12 transactions. (d) Obtained number of sellers when $\beta = 1$, 2, 3, and 5. (e) Obtained number of sellers when $L = 5$, 7, 10, and 15 days. (f) Obtained number of sellers when $\tau = 5$, 7, 10, and 15 days.

Next, we analyse the size of the sliding time window, Δt, used in step 2 of the classification (see Methods in the main text). We compare the results between no sliding time window and with $\Delta t = 10$, 20, and 30 days, as shown in Fig. S3. The number of sellers increases as Δt increases, as expected, but not significantly. The results for different values of Δt are qualitatively equivalent, showing that the classification is robust against the choice of Δt.

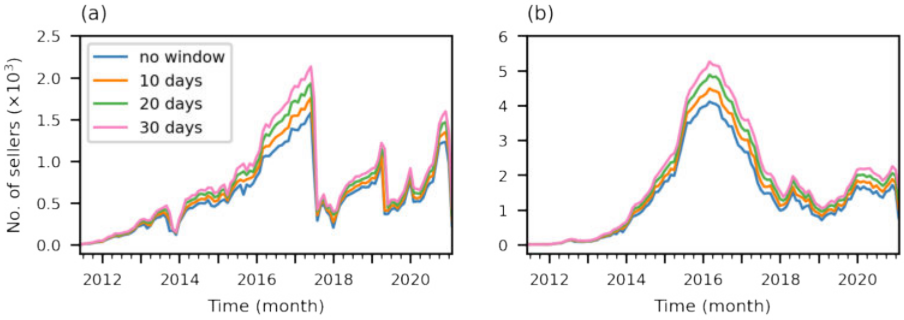

Figure S3: Robustness of the classification of sellers with respect to the sliding time window, Δt. The number of sellers when $\Delta t = 10$, 20, and 30 days, and no sliding time window (no window) is used. (a) Sellers of all markets. (b) Sellers in the U2U network.

Finally, to validate our classification model against an empirical observation, we compare the total number of sellers obtained by our model for a market with official estimates of the number of sellers of that market, which are obtained from difference sources such as web scraping and depositions [1–9]. We collected the number of sellers of nine markets as shown in Table S1. These numbers represent the total number of sellers of the market. Therefore, we compare the total number of sellers obtained by our classification method with that of the official estimate of the market. We conservatively chose the classification parameter values, such that the numbers of sellers obtained by our model underestimate the empirical numbers, as shown in Fig. S4. Moreover, although the robustness tests show that the number of sellers change according to the choice of parameters, our results are qualitatively unchanged.

Table S1: The number of sellers of each market and the source.

Market	Number of sellers	Source
Silk Road	3877	[1]
Evolution	2702	[2]
AlphaBay	40000	[3]
Flugsvamp 2.0	600	[4]
Hansa	8000	[5]
Wall Street	5400	[6]
Empire	4500	[7]
Apollon	1761	[8]
DarkMarket	2400	[9]

S2 Evaluation of the tail exponents of the probability density functions of money received by sellers and sent by buyers

To quantify the heterogeneity in the probability density functions (PDFs) of money received by sellers and sent by buyers in Figs. 2(f) and 2(g) in the main text, we fitted the tails of both distributions with a

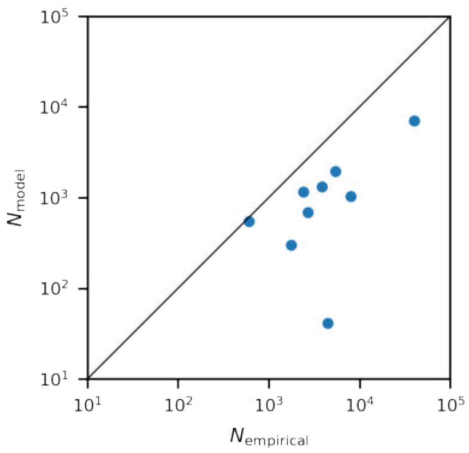

Figure S4: Conservative classification parameters. Comparison between the number of sellers of a market obtained from official sources ($N_{empirical}$) and obtained by our model (N_{model}).

power-law $y = cx^{-\alpha}$. To asses the tail of the distributions, we selected the top 1% of sellers and buyers, which correspond to sellers with total money received greater than 9×10^5 USD and buyers with total money sent greater than 2.5×10^5 USD, respectively. We linearised the power-law equation to $Y = c - \alpha X$, where $Y = \log_{10} y$ and $X = \log_{10} x$. Then, we fitted the linear equation against the real data by minimizing the sum of the squares using the Levenberg–Marquardt algorithm [10]. We obtained exponents in good agreement with the empirical data; for sellers $\alpha = 2.6$ and $c = 3.4 \times 10^7$ with $R^2 = 0.999$, and for buyers $\alpha = 3.2$ and $c = 1.6 \times 10^{10}$ with $R^2 = 0.996$.

S3 Composition of multisellers

Multisellers start to appear by the end of 2013. Their number increases fast, representing more than 20% of all sellers until the first quarter of 2016, as shown in Fig. S5(a). Then, until the end of 2017, their percentage decreases to about 10% of all sellers. After the operation Bayonet, multisellers represent less 3% of sellers.

By definition, multisellers are either market-only sellers or market-U2U sellers. In Fig. S5(b), we show the composition of multisellers as percentages of market-only and market-U2U sellers. Before 2018, the composition oscillates and multisellers are roughly composed by equal parts of the two categories of sellers. During the first three quarter of 2018, more than 80% of multisellers are market-U2U. Then, from the last quarter of 2018 until the end of the observation period, multisellers are composed mostly by market-only sellers.

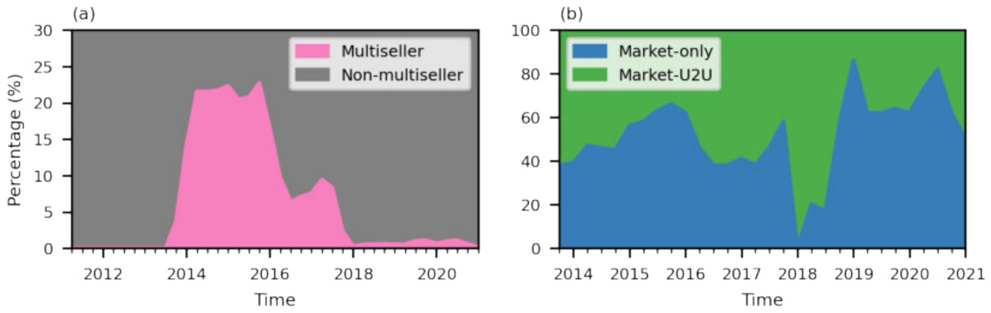

Figure S5: Evolution of the composition of multisellers. (a) Quarterly percentage of multisellers and non-multisellers among all sellers. (b) Quarterly percentage of multisellers that are market-only sellers and market-U2U sellers.

Additionally, multisellers may be active in several markets simultaneously. In Figure S6, we show the monthly number of multisellers that are active in two, three, four, and five markets. We found that five is the maximum number of markets a multiseller was active in our data. Although the number of multisellers active in more than two markets is not negligible, multisellers are predominantly active in two markets only throughout the period of observation. Therefore, we do not distinguish multisellers by the number of markets they operate.

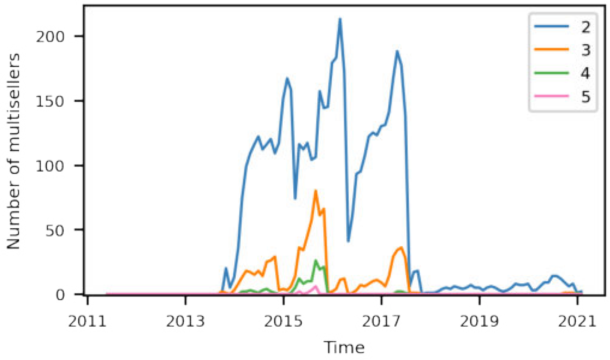

Figure S6: Number of markets that multisellers are present. The monthly number of multisellers active in two (2), three (3), four (4), and five (5) markets.

S4 Impact of operation Bayonet on sellers

To analyse the impact of operation Bayonet on sellers, we measured, for each category and multisellers, the ratio of the number of sellers and the median income relative to respective value before the operation, as shown in Fig. S7. Specifically, we take the quarter when the operation occurred as the time zero, which is in the very beginning if the third quarter of 2017. Therefore, the number of sellers at time zero is the total number of sellers in the quarter before the operation. Then, for each category and multisellers, we compute the ratio between their numbers in subsequent quarters relative to their initial number at time zero, such that 1 corresponds to their number just before the operation. We make the same procedure for the ratio of median.

We observe the impact of the operation with the drop in the number of sellers in all categories and multisellers in the quarters that follows it, as shown if Fig. S7(a). We find that multisellers are the most affected, plummeting by 99% in the end of 2017. After that, the number of multisellers increases but remains notably smaller than it was before the operation. The second most affected are the market-U2U sellers, dropping by 78%. They continue with this tendency, reaching a maximum drop of 89% by the end of the third quarter of 2019. Then, they apparently start to recover by showing a tendency of increase in their number. The market-only sellers drop by 75%, and they recover after that, reaching relatively large numbers by the end of the period of observation. The least affected were the U2U-only sellers, that drop by 42%, also recovering by showing large numbers by the end of the period of observation. Nevertheless, none of them fully recovers after the major shock caused by the operation Bayonet, at least until the end of observed time of the data.

The impact of Bayonet is also reflected on the quarterly median income of sellers, i.e., the median of the money each seller received quarterly, as shown in Fig. S7(b). With the exception of U2U-only sellers, the median income of all types of sellers drops. Similar to what is observed in terms of their number in Fig. S7(a), the most affected were multisellers. Their median income drops by 78% just after the operation. They are followed by market-U2U sellers and market-only sellers, which suffer a drop in their median income of 59% and 47%, respectively. In the following quarters, there is some oscillation with respect to the value before Bayonet, but they all show signs of recovery in the median income, especially from the second half of 2019. Multisellers, specifically, reach median income values higher than that before the operation.

The situation is notably different for U2U-only sellers. Their median income increases by 50% just after operation Bayonet. In fact, we observe a trend of increase in the U2U-only seller median income, reaching

almost six times the value before operation Bayonet by the end of the period of observation. The trend suggests a shift in the ecosystem towards the U2U network after operation Bayonet. This hypothesis is in accordance to the shift in the trading volume of the ecosystem from the markets to the U2U network, as shown in Fig. S8. We computed the quarterly trading volume of all markets and the whole U2U network separately. After the operation Bayonet, the markets' volume decreases, while the U2U network's increases.

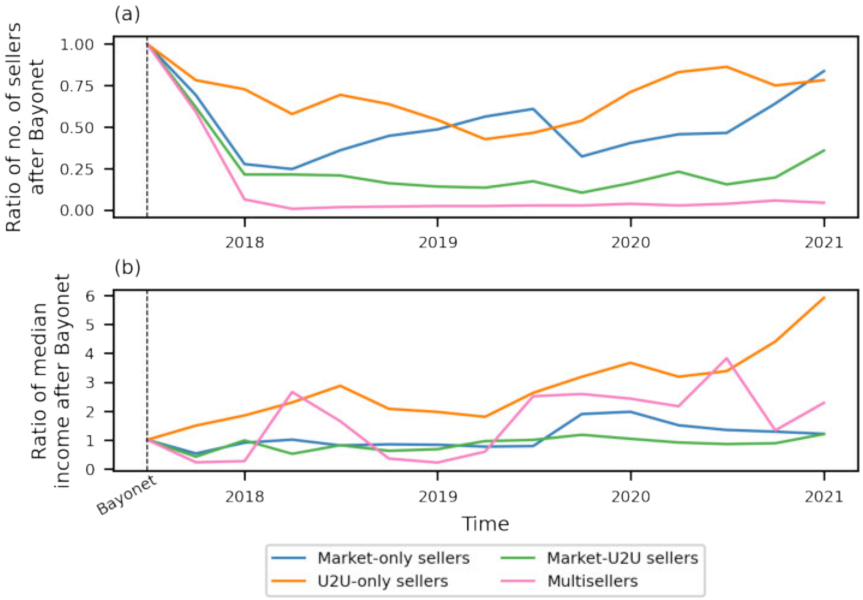

Figure S7: Impact on sellers after operation Bayonet. (a) The quarterly ratio of the number of sellers relative to their number in the quarter before the operation Bayonet. (b) The quarterly ratio of the median income of sellers relative to the median income value in the quarter before the operation Bayonet. In both panels, the vertical dashed line marks the time of the operation Bayonet.

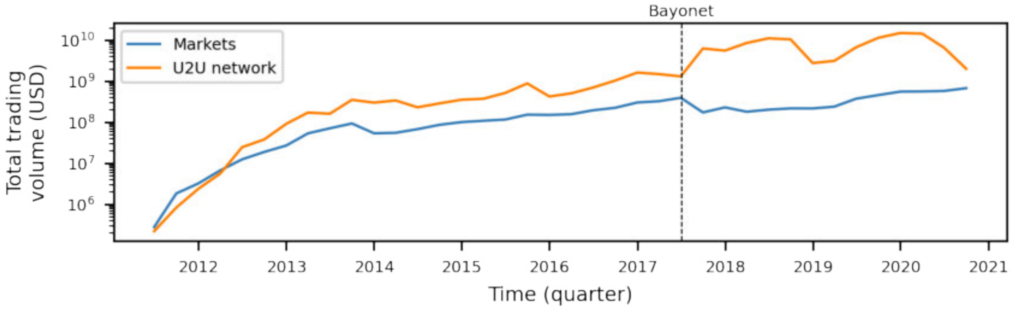

Figure S8: A shift in the trade volume of the ecosystem. The quarterly trading volume in USD computed separately for all markets and for the whole U2U network. The vertical dashed line marks the time of the operation Bayonet, after which, we observe a shift of the trading volume from markets to the U2U network.

S5 Composition and evolution of the S2S network

Until the beginning of 2013, when Silk Road is the dominant market, the giant component of the S2S network is composed mostly by market-only sellers, followed by U2U-only, and market-U2U sellers, as shown in Fig. S9(a). Then, after that period until the end of our observation time, U2U-only is the dominant category in the giant component of the S2S network, followed by market-only, and market-U2U sellers. The

same pattern is observed in the whole S2S network, as shown in Fig. S9(b). Moreover, the same evolution pattern observed in the S2S network giant component (Fig. 6) is also observed in the whole S2S network, as shown in Fig. S10.

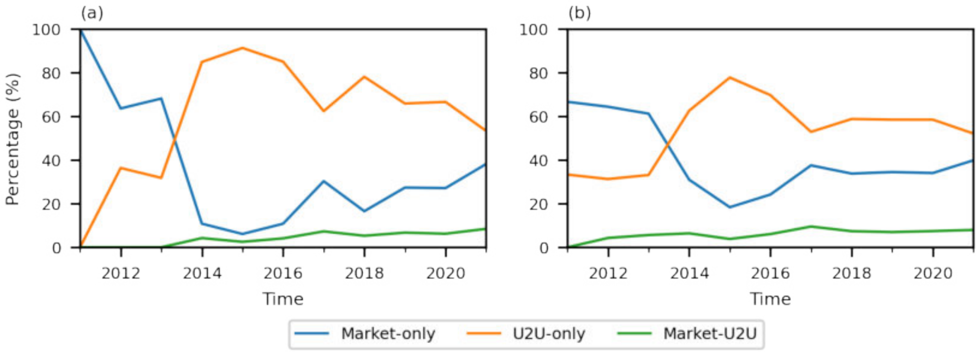

Figure S9: Evolution of the S2S network composition. Percentage of each category of seller in (a) the giant component of the S2S network and (b) the whole S2S network for each year.

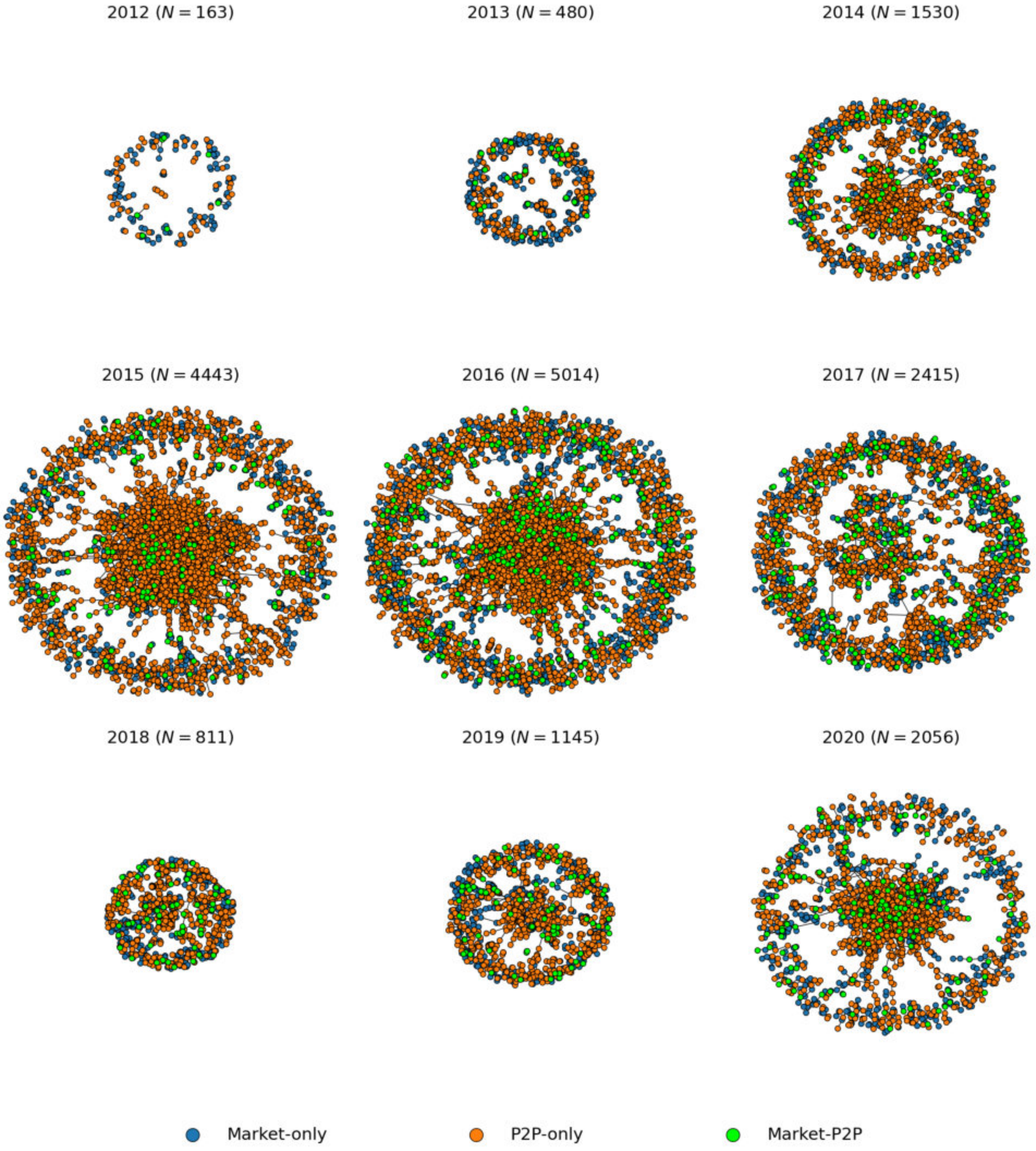

Figure S10: Evolution of the S2S network. The S2S network of U2U transactions between sellers for each year with the respective number of nodes (N). The nodes are sellers that are active in that year, and an edge is placed between two sellers if at least one transaction occurs between them during that year. The whole S2S network shows the same evolution pattern observed in the giant component (Fig. 6).

References

[1] Sealed Complaint 13 MAG 2328: United States of America v. Ross William Ulbricht. https://web.archive.org/web/20140220003018/https://www.cs.columbia.edu/ smb/UlbrichtCriminalComplaint.pdf, 2013. Accessed 18 May, 2023.

[2] D. Rhumorbarbe, L. Staehli, J. Broséus, Q. Rossy, and P. Esseiva. Buying drugs on a darknet market: A better deal? studying the online illicit drug market through the analysis of digital, physical and chemical data. Forensic Sci. Int., 267:173–182, 2016.

[3] US Department of Justice. https://www.justice.gov/opa/pr/alphabay-largest-online-dark-market-shut-down, 2017. Accessed 18 May, 2023.

[4] Presseportal. https://www.presseportal.de/blaulicht/pm/29763/4387169, 2019. Accessed 18 May, 2023.

[5] Netherlands Police. https://web.archive.org/web/20170721082011/, 2017. Accessed 18 May, 2023.

[6] Europol. https://www.europol.europa.eu/media-press/newsroom/news/double-blow-to-dark-web-marketplaces, 2019. Accessed 18 May, 2023.

[7] Webz.io. https://webz.io/blog/dark-web/all-about-empire-market-webz-source-review/, 2020. Accessed 18 May, 2023.

[8] DarkOwl. https://www.darkowl.com/blog-content/apollon-exit-scam-analytical-market-review/, 2020. Accessed 18 May, 2023.

[9] Europol. https://www.europol.europa.eu/media-press/newsroom/news/darkmarket-worlds-largest-illegal-dark-web-marketplace-taken-down, 2021. Accessed 18 May, 2023.

[10] J. J. Moré. The levenberg-marquardt algorithm: implementation and theory. In Numerical analysis: proceedings of the biennial Conference held at Dundee, June 28–July 1, 1977, pages 105–116, 2006.
